# Right Ventricular Response to Acute Hypoxia Exposure: A Systematic Review

**DOI:** 10.3389/fphys.2021.786954

**Published:** 2022-01-12

**Authors:** Argen Mamazhakypov, Meerim Sartmyrzaeva, Nadira Kushubakova, Melis Duishobaev, Abdirashit Maripov, Akylbek Sydykov, Akpay Sarybaev

**Affiliations:** ^1^Department of Internal Medicine, Excellence Cluster Cardio-Pulmonary Institute (CPI), Member of the German Center for Lung Research (DZL), Justus Liebig University of Giessen, Giessen, Germany; ^2^Department of Mountain and Sleep Medicine and Pulmonary Hypertension, National Center of Cardiology and Internal Medicine, Bishkek, Kyrgyzstan; ^3^Kyrgyz Indian Mountain Biomedical Research Center, Bishkek, Kyrgyzstan

**Keywords:** acute hypoxia, high-altitude, right ventricle, pulmonary hypertension, echocardiography

## Abstract

**Background:** Acute hypoxia exposure is associated with an elevation of pulmonary artery pressure (PAP), resulting in an increased hemodynamic load on the right ventricle (RV). In addition, hypoxia may exert direct effects on the RV. However, the RV responses to such challenges are not fully characterized. The aim of this systematic review was to describe the effects of acute hypoxia on the RV in healthy lowland adults.

**Methods:** We systematically reviewed PubMed and Web of Science and article references from 2005 until May 2021 for prospective studies evaluating echocardiographic RV function and morphology in healthy lowland adults at sea level and upon exposure to simulated altitude or high-altitude.

**Results:** We included 37 studies in this systematic review, 12 of which used simulated altitude and 25 were conducted in high-altitude field conditions. Eligible studies reported at least one of the RV variables, which were all based on transthoracic echocardiography assessing RV systolic and diastolic function and RV morphology. The design of these studies significantly differed in terms of mode of ascent to high-altitude, altitude level, duration of high-altitude stay, and timing of measurements. In the majority of the studies, echocardiographic examinations were performed within the first 10 days of high-altitude induction. Studies also differed widely by selectively reporting only a part of multiple RV parameters. Despite consistent increase in PAP documented in all studies, reports on the changes of RV function and morphology greatly differed between studies.

**Conclusion:** This systematic review revealed that the study reports on the effects of acute hypoxia on the RV are controversial and inconclusive. This may be the result of significantly different study designs, non-compliance with international guidelines on RV function assessment and limited statistical power due to small sample sizes. Moreover, the potential impact of other factors such as gender, age, ethnicity, physical activity, mode of ascent and environmental factors such as temperature and humidity on RV responses to hypoxia remained unexplored. Thus, this comprehensive overview will promote reproducible research with improved study designs and methods for the future large-scale prospective studies, which eventually may provide important insights into the RV response to acute hypoxia exposure.

## Introduction

With increasing altitude, the partial pressure of oxygen in the atmosphere falls along with the barometric pressure, leading to a gradually decreasing oxygen availability. Consequently, exposure to high-altitude results in diminished arterial blood oxygen content and initiates acclimatization processes consisting in a series of physiological responses of various organs and systems to maintain an adequate oxygen delivery to the tissues. Particularly, major changes occur in the cardiovascular system. While, the systemic vascular resistance may increase only up to 20%, there is more significant elevation of pulmonary artery pressure (PAP) and pulmonary vascular resistance (PVR) due to hypoxic pulmonary vasoconstriction (HPV), against which the right ventricle (RV) should maintain its contractility (Baggish et al., 2014). HPV is a remarkable cardiovascular response to high-altitude hypoxia exposure (Sydykov et al., 2021). Although, the main purpose of the HPV is to maintain local ventilation-perfusion matching and improve blood oxygenation, in the setting of global lung hypoxia during high-altitude exposure, diffuse HPV results in PAP and PVR increase (Swenson, 2013). The degree of the PAP elevation is usually mild-to-moderate and is not associated with serious adverse health consequences (Sydykov et al., 2021). If exposure to hypoxia persists, PAP may remain elevated (Hilty et al., 2016; Gaur et al., 2021) or become partially attenuated over the course of 2–3 weeks (Ghofrani et al., 2004; Baggish et al., 2010). However, a certain proportion of the population displays an exaggerated HPV (B?rtsch and Gibbs, 2007), which may predispose to high altitude pulmonary edema (HAPE), a potentially life-threatening condition that typically occurs in otherwise healthy individuals after rapid ascent to high-altitude (Sydykov et al., 2021). Importantly, alleviation of HPV by an immediate descent leads to a full recovery from HAPE.

A fall in arterial blood oxygen content initiates cardiac responses, including cardiac output increase, in order to maintain adequate oxygen delivery to the tissues (Naeije, 2010). The cardiac output enhancement observed upon high-altitude exposure is explained mainly by increased heart rate with maintained or decreased stroke volume (Naeije, 2010). In high-altitude hypoxia conditions with decreased oxygen availability, augmented cardiac output along with the enhanced load to the RV due to elevated PVR increases myocardial oxygen demand, thus predisposing the RV to myocardial dysfunction. In addition, cardiac performance may be hampered by various factors associated with the high-altitude exposure, such as increased blood viscosity, sympathetic nervous system activation and hypovolemia (Naeije, 2010). Thus, the RV not only should maintain or couple its contractility to increased afterload but also withstand other adverse challenges associated with high-altitude. All these factors together require RV functional and geometrical adaptation to maintain cardiac function and oxygen delivery to the tissues.

Available studies that evaluated the effects of acute high-altitude exposure on the RV produced conflicting results (Naeije and Dedobbeleer, 2013; Richalet and Pichon, 2014). While some studies demonstrated an augmented RV function in response to acute hypoxia-induced afterload increase (Berger et al., 2018; Sareban et al., 2020), others found no change (Huez et al., 2005) or even an impaired RV function (Reichenberger et al., 2007; Hanaoka et al., 2011; Page et al., 2013). Furthermore, there is anecdotal evidence of acute RV failure due to increased RV pressure overload in a healthy individual upon high-altitude exposure (Huez et al., 2007). These discrepancies in the existing literature are poorly understood but might be related to the considerable differences in methods and research designs between the studies and lack of compliance with the guidelines for the assessment of the RV function and morphology (Rudski et al., 2010). In addition, gender (Boos et al., 2016), age (Stewart et al., 2020), ethnicity (Gaur et al., 2021), individual susceptibility (Huez et al., 2007) and some other factors can impact RV performance upon high-altitude hypoxia exposure. Further, at high altitude, RV might be subjected to hemodynamic load of variable severity in different individuals due to great inter-individual variability in the HPV.

In lowlanders, prolonged high-altitude stay is associated with sustained HPV and pulmonary vascular remodeling (Groves et al., 1987; Maggiorini et al., 2001; Luks et al., 2017). Depending on their severity, these changes can cause persistent elevation of PAP and PVR resulting in increased RV afterload, which may lead to RV remodeling and dysfunction (Yang et al., 2015). During prolonged stay at extreme high-altitudes (5800-6700 m), some healthy lowlanders might develop severe RV failure with peripheral congestion syndrome, which is fully reversible upon returning to low altitude (Anand et al., 1990). However, it remains unknown whether acute RV dysfunction affects subsequent development of RV remodeling and failure with prolonged stay at high-altitude.

Although right heart catheterization is the gold standard method for the assessment of pulmonary hemodynamics and RV function, its application in healthy humans in high-altitude settings is challenging due to logistics (remote area) and its invasive nature. Alternatively, echocardiography has become a widely available and reliable method to study cardiovascular physiology (Soria et al., 2016, 2019). It has been proven to be a valuable tool to characterize RV function and morphology in various cardiovascular conditions (Taleb et al., 2013; Greiner et al., 2014). Importantly, echocardiography allows measuring cardiac function serially over the specified time periods and altitude levels (Galie et al., 2016). Consequently, echocardiography has become a widely available and reliable method to study cardiovascular physiology in high-altitude research (Fagenholz et al., 2012; Feletti et al., 2018).

Recently emerged state-of-the-art echocardiography imaging modalities such as speckle-tracking and 3D echocardiography have significantly improved our understanding of the left ventricular (LV) physiology in lowlanders acutely exposed to high-altitude and in permanent high-altitude residents (Stembridge et al., 2015, 2016, 2019). However, these techniques remain underutilized for the in-depth characterization of the RV function and morphology in high-altitude settings. Understanding the physiology of the RV responses to acute hypoxia is of significant importance for gaining insights into mechanisms underlying acute RV dysfunction and failure in various cardiopulmonary diseases. Thus, this systematic review aims to evaluate the RV responses to acute simulated altitude exposure and high-altitude ascent in healthy lowland individuals.

## Methods

### Data Sources and Search Strategy

A comprehensive literature search for the relevant articles was performed between January 2005 and May 2021 using online databases PubMed and Web of Science. The aim was to identify studies investigating the effects of acute simulated altitude or high-altitude exposure on RV systolic and diastolic function and morphologic parameters in healthy lowland adults. The search strategy was implemented using the following Boolean operators (AND/OR) and terms: “right ventricular function” OR “right ventricular dysfunction” OR “cardiac function” AND healthy subjects OR healthy individuals OR humans AND “echocardiography” OR “ultrasound imaging” AND hypoxia exposure OR “high altitude” OR hypoxia chamber OR hypoxia room. The search was refined by applying the limits “Humans” and “All adult: +19 years” in PubMed and searching within the subject areas of physiology, respiratory system, cardiovascular system and cardiology in the Web of Science. The exact search terms and Boolean operators used in PubMed and Web of Science are provided in the Supplementary Material. Additionally, manual searches were conducted using Google Scholar and reference lists of the eligible articles to retrieve further articles.

### Inclusion and Exclusion Criteria

The inclusion criteria for the relevant studies were: (1) involving healthy lowland subjects aged ≥19 years that had not been exposed to altitudes over 2,500 m in the last 3 weeks before study inclusion; (2) simulated altitude or high-altitude field studies at elevations of 2,500 m and over; (3) reporting at least one of the echocardiographic parameters of the RV systolic or diastolic function or its morphology with numerical values or plots with indicated statistical changes; (4) measurements were performed at baseline (see level with normoxia breathing) and after hypoxia exposure for 15 min up to 1 month; (5) full-text in English; and (6) human subjects. Exclusion criteria were: (1) not reporting measurement results at baseline or after hypoxia exposure; (2) studies with unclear design; (3) case reports, reviews, abstracts, and editorials.

### Study Selection and Data Extraction

Two authors (Argen Mamazhakypov and Meerim Sartmyrzaeva) independently screened the retrieved articles by titles and abstracts according to the inclusion and exclusion criteria. Then, the full-texts of the relevant articles were obtained, and the same two authors independently screened the full-text of the articles. Disagreements were resolved by discussions between all the authors.

Two authors (Argen Mamazhakypov and Meerim Sartmyrzaeva) independently extracted the following data (Tables 1, 2): publication details (authors, publication year), sample characteristics (sample size, gender, age), hypoxia exposure profile (simulated altitude or high-altitude field study, hypoxia exposure duration, altitude elevation), mode of ascent (staged acclimatization or without pre-acclimatization), and summarized the outcome measures (RV function and morphology parameters) and the changes in RV parameters upon simulated altitude or high-altitude exposure (**Tables 4**, **6**).

**Table 1:** Summary of the studies evaluating right ventricular function and morphology upon acute simulated altitude exposure.

**Study**	**Sample size (M/F)**	**Age (mean ± SD or mean (range) years)**	**Health status**	**Type of hypoxia exposure**	**Baseline altitude (m)**	**FiO_**2**_ (%) or altitude level (m)**	**Rate of exposure**	**Hypoxic duration**	**Pulmonary hemodynamics parameters**	**RV diastolic functional parameters**	**RV systolic functional parameters**	**RV morphology parameters**
Huez et al., 2005	25 (14/11)	32 ± 8	Healthy	Hypoxic breathing	Brussels (13)	FiO_2_−12% (≈4,500)	NA	90 min	TRG, PAAT/ET	RV E/A, RV E′, RV A′, RV E′/A′	TAPSE, RV-S′	RV-EDA
Kjaergaard et al., 2007	14 (13/1)	33 (21–55)	Healthy	Hypoxic breathing	NA	FiO_2_−12.5%	NA	1 h	sPAP, PAAT, PVR	RV-E′, RV-A′	TAPSE, RV-MPI, RV-S′, RV-FAC	RV-EDA
Reichenberger et al., 2007	14 (12/2)	37 (23–55)	Healthy	Hypoxia breathing	Giessen (171)	FiO_2_−10% (≈5,500)	NA	2 h	sPAP, PAAT/ET		TAPSE, RV-MPI	
(HAPE-s) (Hanaoka et al., 2011)	11 (11/0)	50.9 ± 13.2	Healthy	Hypoxic breathing	Matsumoto (610)	4,000	NA	30 min	sPAP		RV-MPI	
(HAPE-r) (Hanaoka et al., 2011)	9 (9/9)	53.1 ± 13.4	Healthy	Hypoxic breathing	Matsumoto (610)	4,000	NA	40–45 min	sPAP		RV-MPI	
Pavelescu and Naeije, 2012	10 (7/3)	24 ± 3	Healthy	Hypoxic breathing	NA	FiO_2_−12%	NA	2 h	sPAP, TRG, PAAT/ET, PVR	RV-E/A, RV-E′, RV-A′, RV-E′/A′	TAPSE, RV-S′, RV-FAC	RV-EDA
Goebel et al., 2013	14 (12/2)	NA (25–41)	Healthy	Normobaric hypoxia chamber	NA	FiO_2_−9.9%	2h → 4,000 m (4 h) → 5,500	2 h	TRG, PVR	RV-E/A	TAPSE, RV-GLS	
Boos et al., 2013	14 (14/0)	30.5 ± 4.3	Healthy	Hypobaric hypoxia chamber	Henlow (40)	4,800	1,219 m per min	>150 min	sPAP, PAAT,	RV-E, RV-A, RV-E/A, RV-E′, RV-A′	TAPSE, RV-MPI, RV-S′	
(Women) (Boos et al., 2016)	7 (0/7)	25.9 ± 3.2	Healthy	Normobaric hypoxia chamber	Leeds (113)	FiO_2_−11.4% (≈4,800)	NA	>150 min	sPAP, PAAT, PVR		TAPSE	
(Men) (Boos et al., 2016)	7 (7/0)	27.3 ± 4.4	Healthy	Normobaric hypoxia chamber	Leeds (113)	FiO_2_−11.4%	NA	>150 min	PAAT, sPAP, PVR		TAPSE	
Seccombe et al., 2017	7 (4/3)	51 ± 15	Healthy	Hypoxic breathing		FiO_2_−15%	NA	20 min	sPAP		TAPSE, RV-MPI, RV-FAC	RV-ESA
Netzer et al., 2017	35 (24/11)	NA	Healthy	Normobaric hypoxia chamber	Bad Aibling (492)	FiO_2_−11%	NA	30, 60, 100, 150 min	sPAP, PAAT	RV-E′, RV-A′	TAPSE, RV-MPI, RV-S′	RV-EDA, RVD1, RVD2, RVD3
Pezzuto et al., 2018	17 (10/7)	24 ± 6	Healthy	Hypoxic breathing	NA	FiO_2_−12%	NA	15, 30, 45, 60 min	mPAP, PVR		TAPSE, RV-S′, RV-FAC	RV-EDA
Ewalts et al., 2021	15 (12/3)	25 ± 4	Healthy	Hypoxic breathing	Cardiff (17)	FiO_2_−12%	NA	30 min	sPAP, mPAP, PVR		RV-FAC, RV-GLS	RV-EDA, RV-ESA

*TRG, tricuspid regurgitation gradient; sPAP, systolic pulmonary artery pressure; mPAP, mean pulmonary artery pressure; PAAT, pulmonary artery acceleration time; PAAT/ET, pulmonary acceleration time to ejection time ratio; PVR, pulmonary vascular resistance; RV-E, tricuspid inflow early diastolic velocity; RV-A, tricuspid inflow late diastolic velocity; RV-E/A, tricuspid inflow early diastolic velocity to late diastolic velocity; RV-E′, tricuspid annulus early diastolic velocity by tissue Doppler imaging; RV-A′, tricuspid annulus late diastolic velocity by tissue Doppler imaging; RV-E′/A′, tissue Doppler early and late diastolic tricuspid velocities ratio; RV-S′, peak systolic velocity with tissue Doppler imaging at the tricuspid annulus; TAPSE, tricuspid annular plane systolic excursion, RV-MPI, right ventricular myocardial performance index; RV-FAC, right ventricular fractional area change; RV-EDA, right ventricular end-diastolic area; RV-ESA, right ventricular end-systolic area; RVD1, right ventricular end-diastolic basal cavity dimension; RVD2, right ventricular end-diastolic mid cavity dimension; RVD3, right ventricular end-diastolic longitudinal dimension; RV-GLS, right ventricular global longitudinal strain*.

**Table 2:** Summary of the studies evaluating right ventricular function and morphology upon acute high-altitude exposure.

**Study**	**Sample size (M/F)**	**Age (mean ± SD or mean (range) years)**	**Health status**	**Baseline altitude (m)**	**High altitude location (altitude in m)**	**Ascent mode/duration of ascent**	**High altitude measurement timepoints**	**Pulmonary hemodynamics parameters**	**RV diastolic functional parameters**	**RV systolic functional parameters**	**RV structural parameters**
Huez et al., 2009	15 (7/8)	36 ± 12	Healthy	Brussels (13)	La Paz (3,750), Huayna Potosi (4,850 m)	Direct/24 h	1st day (3,750 m), 10th day (4,850 m)	TRG, PAAT, mPAP	RV-E, RV-A, RV-E′, RV-A′, RV-E′/A′	TAPSE, RV-S′, RV-MPI	
de Vries et al., 2010	7 (4/3)	41 ± 16	Healthy	Zwolle (4)	Aconcagua (4,200)	Staged/10-day trip	10th day		RV-E′	TAPSE	
Pratali et al., 2010	18 (10/8)	45 ± 10	Healthy	Aosta (583)	Namche Bazaar (3,440), Gokyo (4,790), Gorak shep (5,130)	Staged/6 days	6th day (3,440 m), 10th day (4,790 m), 14th day (5,130 m)	sPAP		TAPSE	
Page et al., 2013	14 (8/6)	46 ± 12.4	Healthy	Montreal (30)	Namche Bazar (3,450), Chukkung (4,730)	Staged/4 day	4th and 7th days	sPAP, PVR	RV-E/A	TAPSE, RV-S′, RV-MPI, RV-GLS, RV-FWLS	
Boos et al., 2014	19 (10/9)	35.4 ± 8.3	Healthy	NA (1,300)	Namche Bazar (3,440), Pheriche (4,270), Gorak Shep (5,150)	Staged/2 days	2nd day (3,440 m), 6th day (4,270 m), 9th day (5,150 m)	sPAP	RV-E, RV-E′	RV-S′	
Stembridge et al., 2014	9 (9/0)	34 ± 7	Healthy	Kelowna (344)	Lobuche (5,050)	Staged/10-day trip	10th day	sPAP	RV-E′, RV-A′	TAPSE, RV-S′	RV-EDA, RV-ESA
Dedobbeleer et al., 2015	25 (13/12)	31 ± 13	Healthy	Brussels (13)	Cerro de Pasco (4,350)	Staged/4 days	4th day	TRG, mPAP, PVR		TAPSE, RV-S′, RV-FAC, RV-MPI	
Hilty et al., 2016	7 (7/0)	26.6 ± 1.4	Healthy	Zurich (488)	Jungfraujoch (3,454)	Direct/24 h (train)	2nd, 10th, 18th, and 26th days	sPAP, PVR, PAAT		TAPSE, RV-FAC, RV-MPI	RV-EDA
De Boeck et al., 2018	23 (15/8)	43 ± 9	Healthy	Zurich (450)	Margherita hut (4,559)	(car, climb) (1 night at 3,647 m)	2nd, 3rd, and 4th days	TRG		TAPSE, RV-S′, RV-FAC	RV-EDA, RV-ESA
Maufrais et al., 2017	11 (11/0)	28 ± 8	Healthy	Grenoble (212)	Mont Blanc (4,350)	Direct/3 ± 2 h (helicopter)	0.5th, 2nd, 4th, and 6th days	sPAP, TRG, PVR	RV-E, RV-A, RV-E/A, RV-E′, RV-A‘	TAPSE, RV-S′, RV-FAC, RV-FWLS	RV-EDA, RV-ESA
Qiu et al., 2017	123 (123/0)	23.77 ± 4.5	Healthy	Chengdu (500)	Lhasa (3,700)	Direct/2 h (airplane)	1st day	mPAP		RV-MPI	
Berger et al., 2018	17 (11/6)	36 ± 12	Healthy	Salzburg (423)	Monte Rosa (4,559)	20 h (car, climb), 1 night at 3,611 m	7th, 20th, 32nd, 44th h	sPAP		TAPSE, RV-S′, RV-FAC, RV-MPI	
Hilty et al., 2019	41 (22/19)	45.8 ± 11.9	Healthy	Bern (553)	C2 (6,022 m), C3 (7,042)	Staged/1 week (by foot)	8th (*n* = 36), and 15th (*n* = 15) days	TRG		TAPSE	
Maufrais et al., 2019	20 (29/0)	39 ± 16	Healthy	Bangor (65)	Larkyu (5,085)	Staged/10-day trip	10th day	sPAP	RV-E, RV-A, RV-E/A, RV-E‘, RV-A′	TAPSE, RV-S′, RV-FAC	
Stembridge et al., 2019	12 (12/0)	27 ± 6	Healthy	Kelowna (344)	Barcroft Laboratory (3,800)	Direct (9–10 h in motor vehicle)	5–10th days	sPAP, PVR	RV-E, RV-A, RV-E/A	RV-FAC, RV-GLS	RV-EDA, RV-ESA
(Young) (Stewart et al., 2020)	14 (8/6)	32 ± 5	Healthy	Moshi (880)	Shira (3,100), Barafu (4,800)	Staged/3 days	3rd (3,100 m), and 8th (4,800 m) days	RVSP, mPAP, PVR	RV-E′, RV-A′, RV-E′/A′	TAPSE, RV-S′, RV-FAC	RV-EDA, RV-ESA
(Old) (Stewart et al., 2020)	13 (8/5)	59 ± 5	Healthy	Moshi (880)	Shira (3,100), Barafu (4,800)	Staged/3 days	3rd (3,100 m), 8th (4,800 m) days	RVSP, mPAP, PVR	RV-E′, RV-A′, RV-E′/A′	TAPSE, RV-S′, RV-FAC	RV-EDA, RV-ESA
Tian et al., 2020	240 (240/0)		Healthy	Chongqing (500)	Litang (4,100)	Staged/7 days (bus)	5th day	sPAP, mPAP, PAAT/ET	RV-E, RV-A, RV-E/A, RV-E′	RV-S′, RV-FAC, RV-MPI	RV-EDAI, RV-ESAI
Yang et al., 2020b	121 (121/0)	20 (19, 21)	Healthy	Chongqing (400)	Litang (4,100)	Staged/7 days (bus)	1st day	sPAP, mPAP, PAWP, PVR	RV-E/A, RV-E′	RV-S′, RV-FAC	RV-EDA, RV-ESA
Yang et al., 2020a	108 (108/0)	20 (19, 22)	Healthy	Chongqing (400)	Litang (4,100)	Staged/7 days (bus)	1st day	sPAP, PAAT, PAAT/ET, mPAP	RV-E/A, RV-E‘	TAPSE, RV-S′, RV-FAC, RV-GLS	RV-EDA, RV-ESA
Ewalts et al., 2021	10 (10/0)	27 ± 6	Healthy	Kelowna (344)	Barcroft Laboratory (3,800)	Direct (9–10 h in motor vehicle)	5–10th days	TRV, sPAP, mPAP, PVR		RV-FAC, RV-GLS	RV-EDA, RV-ESA
Sareban et al., 2020	50 (50/0)	36 ± 11	Healthy	Sulzburg (424)	Monte Rosa (4,559)	20 h (car, climb) (1 night at 3,611 m)	7th, 20th, 44th h	sPAP		TAPSE, RV-S′, RV-FAC, RV-MPI, RV-GLS	RV-EDA, RV-ESA, RVD1, RVD2, RVD3
(Indians) (Gaur et al., 2021)	10 (10/0)	23.8 ± 2.1	Healthy	Bishkek (800)	Sook Pass (4,111)	Direct/3 h (car)	3rd, 7th, 14th, 21st days	PVR, TRG	RV-E, RV-A, RV-E/A, RV-E′, RV-A′, RV-E′/A′	TAPSE, RV-S′, RV-MPI	RVD1, RVD2, RVD3
(Kyrgyz) (Gaur et al., 2021)	20 (20/0)	22.6 ± 2.1	Healthy	Bishkek (800)	Sook Pass (4,111)	Direct/3 h (car)	3rd, 7th, 14th, 21st days	PVR, TRG	RV-E, RV-A, RV-E/A, RV-E′, RV-A′, RV-E′/A′	TAPSE, RV-S′, RV-MPI	RVD1, RVD2, RVD3
He et al., 2021	82 (82/0)	20 (19–21)	Healthy	Anggongqiao (400)	Litang (4,100)	Direct/Staged/7-day trip	5 ± 2 h	sPAP, TRG	RV-E, RV-A, RV-E/A, RV-E′, RV-A′, RV-E′/A′		RV-EDAI, RV-ESAI
Lichtblau et al., 2021	21 (8/13)	25 (21–28)	Healthy	Chile (520)	ALMA (5,050)	Staged/Car, walk, 1 night at 2,900 m	4–8 h	sPAP, TRG, PVR		TAPSE, RV-FAC	
Yuan et al., 2021	99 (69/30)	25 (21.3, 29)	Healthy	Chengdu (500)	Litang (4,100)	Staged/2 days (bus)	1st day	sPAP, mPAP, PAAT, PAAT/ET, PVR, PAWP	RV-E, RV-A, RV-E/A, RV-E′	TAPSE, RV-S′, RV-FAC, RV-GLS	RV-EDA, RV-ESA, RVD1, RVD2

*TRG, tricuspid regurgitation gradient; sPAP, systolic pulmonary artery pressure; mPAP, mean pulmonary artery pressure; PAAT, pulmonary artery acceleration time; PAAT/ET, pulmonary acceleration time to ejection time ratio; PAWP, pulmonary artery wedge pressure; PVR, pulmonary vascular resistance; RV-E, tricuspid inflow early diastolic velocity; RV-A, tricuspid inflow late diastolic velocity; RV-E/A, tricuspid inflow early diastolic velocity to late diastolic velocity; RV-E′, tricuspid annulus early diastolic velocity by tissue Doppler imaging; RV-A′, tricuspid annulus late diastolic velocity by tissue Doppler imaging; RV-E′/A′, tissue Doppler early and late diastolic tricuspid velocities ratio; RV-S′, peak systolic velocity with tissue Doppler imaging at the tricuspid annulus; TAPSE, tricuspid annular plane systolic excursion, RV-MPI, right ventricular myocardial performance index; RV-FAC, right ventricular fractional area change; RV-EDA, right ventricular end-diastolic area; RV-ESA, right ventricular end-systolic area; RV-EDAI, right ventricular end-diastolic area index; RV-ESAI, right ventricular end-systolic area index; RVD1, right ventricular end-diastolic basal cavity dimension; RVD2, right ventricular end-diastolic mid cavity dimension; RVD3, right ventricular end-diastolic longitudinal dimension; RV-GLS, right ventricular global longitudinal strain*.

## Results

### Study Selection

**Figure 1:**
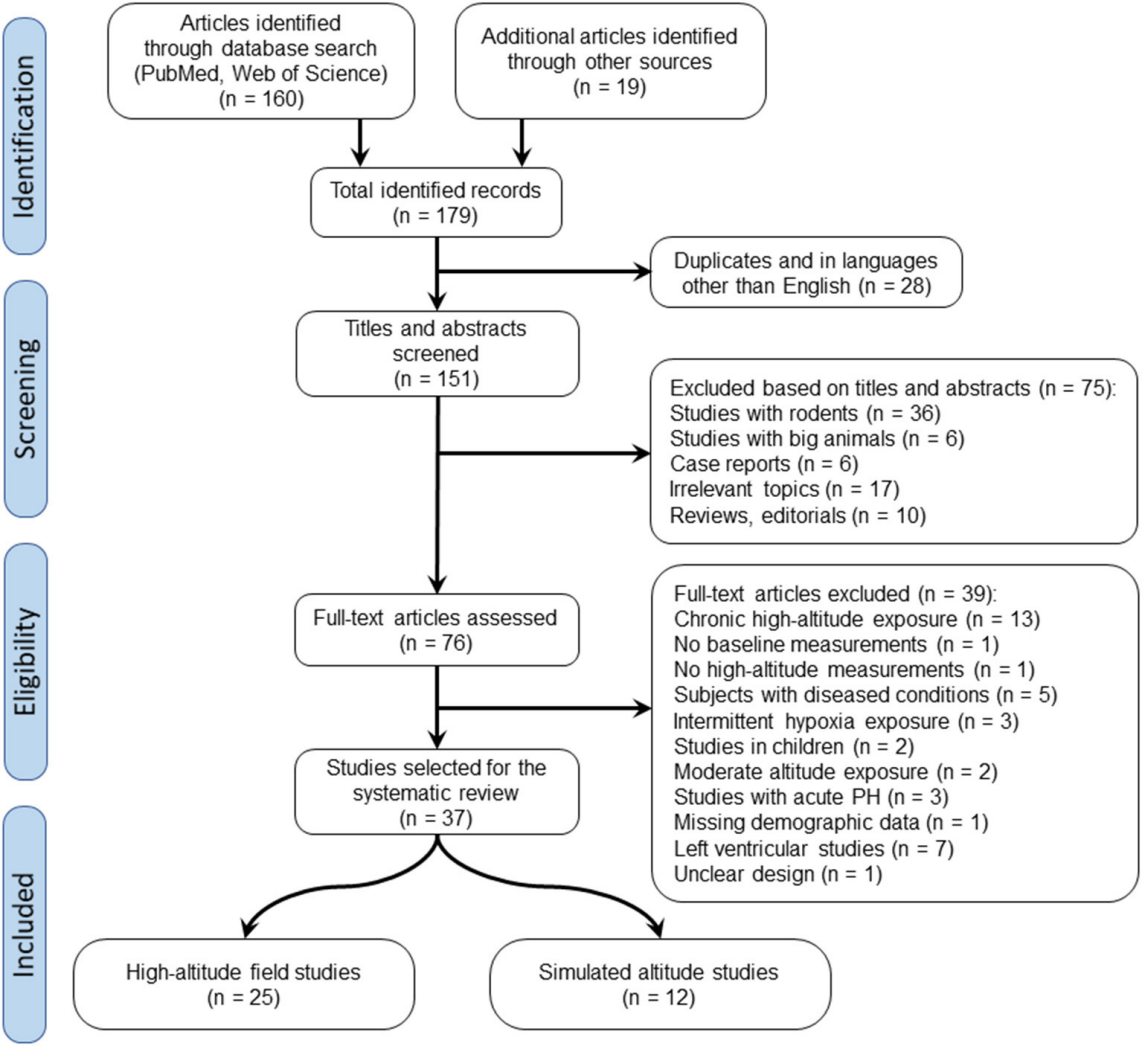
Flow chart representation of literature search.

Using electronic searches of the databases PubMed and Web of Science, the initial search retrieved 160 records. Additional 19 articles were identified through other sources. After excluding duplicates and articles in languages other than English, we screened the remaining 151 articles by their title and abstract and excluded animal experiments, case reports, review articles, editorials, conference abstracts and articles on irrelevant topics. The remaining 76 records were evaluated for eligibility by full-text assessment, of which 37 studies fulfilled the inclusion criteria and thus were selected for systematic review. The selection process is shown in the PRISMA flow diagram in Figure 1.

### Study Characteristics

The characteristics of the selected 37 studies are summarized in Tables 1, 2. The studies differed in their design (e.g., mode of ascent, altitude level, and measurement time-points at high-altitude), simulated altitude characteristics (e.g., hypoxia breathing, normobaric or hypobaric hypoxia chamber exposure), reported RV parameters, and subject characteristics (number, gender, and age). The marked methodological heterogeneity across these studies and the small number of papers prevented meta-analysis. Hence, we provide a descriptive analysis of the results.

According to the mode of hypoxia exposure the selected studies were divided into simulated altitude and high-altitude field studies. Twelve studies exploited simulated altitude using normobaric or hypobaric hypoxia chambers or hypoxia breathing, and 25 studies were conducted in high-altitude field conditions.

### Effects of Simulated Altitude on the Right Ventricle

Twelve studies explored effects of simulated altitude and included a total of 199 healthy adults (Huez et al., 2005; Kjaergaard et al., 2007; Reichenberger et al., 2007; Hanaoka et al., 2011; Pavelescu and Naeije, 2012; Boos et al., 2013, 2016; Goebel et al., 2013; Netzer et al., 2017; Seccombe et al., 2017; Pezzuto et al., 2018; Ewalts et al., 2021). Study participants were exposed to various degrees of hypoxia from 9.9 to 12.5% fraction of inspired oxygen (FiO_2_), which correspond to altitude levels ranging from 4,000 to 5,500 m. Altitude was simulated by either hypobaric or normobaric hypoxic chamber exposure or hypoxia breathing. Duration of the hypoxia exposure varied from 15 to 150 min. In the majority of the studies, a single echocardiography investigation was performed at high altitude, but in few of them, serial investigations were done. Echocardiographic parameters assessed systolic and diastolic RV function and RV morphology. The summary of the assessed RV parameters is presented in Table 3 and the changes of the parameters upon hypoxia exposure are summarized in Table 4.

**Table 3:** Echocardiographic parameters assessed in acute simulated altitude studies.

**Studies**	**Diastolic function**						**Systolic function**					**Morphology**				
	RV-E	RV-A	RV-E/A	RV-E′	RV-A′	RV- E′/A′	RV-S′	TAPSE	RV-FAC	RV-MPI	RV-GLS	RVD1	RVD2	RVD3	RV-EDA	RV-ESA
Huez et al., 2005			✓	✓	✓	✓	✓	✓							✓	
Kjaergaard et al., 2007				✓	✓		✓	✓	✓	✓					✓	
Reichenberger et al., 2007								✓		✓						
Hanaoka et al., 2011										✓						
Pavelescu and Naeije, 2012			✓	✓	✓	✓	✓	✓	✓							
Goebel et al., 2013			✓					✓								
Boos et al., 2013	✓	✓	✓	✓			✓	✓		✓						
Boos et al., 2016								✓								
Seccombe et al., 2017								✓	✓	✓						
Netzer et al., 2017				✓	✓		✓	✓		✓		✓	✓	✓	✓	
Pezzuto et al., 2018							✓	✓	✓						✓	
Ewalts et al., 2021									✓		✓				✓	✓

*✓ Indicates that the echocardiographic parameter was provided in the corresponding studies; empty cell indicate that the echocardiographic parameter is not provided in the corresponding studies*.

**Table 4:** Summary of the studies evaluating the impact of simulated altitude exposure on the right ventricle.

**Study**	**Subjects (n)**	**Measurement points (minutes or hours)**	**FiO_**2**_ (%)**	**Equivalent altitude (m)**	**Systolic function parameters**	**Diastolic function parameters**	**Morphology parameters**
Huez et al., 2005	25	90 min	12	4,500	↔ RV-FS, ↔ TAPSE, ↔ RV-S′	↓ RV-E/A, ↔ RV-E′, ↑ RV-A′, ↓ RV-E′/A′	↔ RV-EDA,
Kjaergaard et al., 2007	14	1 h	12.5	4,200	↑ TAPSE, ↔ RV-FAC, ↔ RV-S′, ↔ RV-MPI	↔ RV-E′, ↔ RV-A′	↔ RV-EDA,
Reichenberger et al., 2007	14	2 h	10	4,500	↑ RV-MPI, ↓ TAPSE		
(HAPE-s) (Hanaoka et al., 2011)	11	30 min	NA	4,000	↑ RV-MPI		
(HAPE-r) (Hanaoka et al., 2011)	9	30 min	NA	4,000	↔ RV-MPI		
Pavelescu and Naeije, 2012	10	2 h	12	4,500	↔ RV-FAC, ↔ TAPSE, ↔ RV-S′	↓ RV-E/A, ↔ RV-E′, ↑ RV-A′, ↓RV-E′/A′	↔ RV-EDA
Goebel et al., 2013	14	2 h	9.9	5,500	↔ TAPSE, ↑ RV-strain		
Boos et al., 2013	14	≥150 min	11.4	4,800	↔ TAPSE, ↑ RV-S′, ↔ RV-MPI	↔ RV-E, ↔ RV-A, ↔ RV-E/A, ↔ RV-E′, ↑ RV-A′	
(Females) (Boos et al., 2016)	7	≥150 min	11.4	4,800	↔ TAPSE		
(Males) (Boos et al., 2016)	7	≥150 min	11.4	4,800	↔ TAPSE		
Seccombe et al., 2017	7	20 min	15	2,500	↔ RV-FAC, ↔ RV-MPI, ↔ TAPSE		↔ RV-EDA, ↔ RV-ESA
Netzer et al., 2017	35	30 min	10	4,500	↔ RV-MPI, ↔ TAPSE, ↔ RV-S′	↔ RV-E′, ↔ RV-A′	↑ RVD1, ↑ RVD2, ↑ RVD3, ↑ RV-EDA
		60 min	10	4,500	↔ RV-MPI, ↓ TAPSE, ↓ RV-S′	↔ RV-E′, ↔ RV-A′	↑ RVD1, ↑ RVD2, ↑ RVD3, ↑ RV-EDA
		100 min	10	4,500	↑ RV-MPI, ↓ TAPSE, ↓ RV-S′	↔ RV-E′, ↔ RV-A′	↑ RVD1, ↑ RVD2, ↑ RVD3, ↑ RV-EDA
		150 min	10	4,500	↑ RV-MPI, ↓ TAPSE, ↓ RV-S′	↔ RV-E′, ↔ RV-A′	↑ RVD1, ↑ RVD2, ↑ RVD3, ↑ RV-EDA
Pezzuto et al., 2018	17	15 min	12	4,500	↔ TAPSE, ↔ RV-S′, ↔ RV-FAC,		↔ RV-EDA
		30 min	12	4,500	↔ TAPSE, ↔ RV-S′, ↔ RV-FAC,		↔ RV-EDA
		45 min	12	4,500	↔ TAPSE, ↔ RV-S′, ↔ RV-FAC,		↔ RV-EDA
		60 min	12	4,500	↔ TAPSE, ↔ RV-S′, ↔ RV-FAC,		↔ RV-EDA
Ewalts et al., 2021	15	30 min	12	45,00	↔ RV-FAC, ↔ RV-SV, ↔ RV-GLS, ↔ RV-GLSR		↔ RV-EDA, ↔ RV-ESA

*↑, high-altitude values of the measured parameters are significantly increased compared to baseline values; ↓, high-altitude values of measured parameters are significantly decreased compared to baseline values; ↔ high-altitude values of measured parameters are not changed significantly compared to baseline values*.

#### Right Ventricular Systolic Function

In all of the studies, at least one of the following RV systolic function parameters was reported: tricuspid annular plane systolic excursion (TAPSE), RV fractional area change (RV-FAC), tissue Doppler-derived peak tricuspid annulus systolic velocity (RV-S′), RV myocardial performance index (RV-MPI) and RV global longitudinal strain (RV-GLS). The most frequently measured RV systolic parameter was TAPSE, which was evaluated in 10 studies (Huez et al., 2005; Kjaergaard et al., 2007; Reichenberger et al., 2007; Pavelescu and Naeije, 2012; Boos et al., 2013, 2016; Goebel et al., 2013; Netzer et al., 2017; Seccombe et al., 2017; Pezzuto et al., 2018). In 7 of these studies, TAPSE values remained unchanged upon exposure to acute hypoxia (Huez et al., 2005; Pavelescu and Naeije, 2012; Boos et al., 2013, 2016; Goebel et al., 2013; Seccombe et al., 2017; Pezzuto et al., 2018). One study reported significant elevation of TAPSE following 1 h of hypoxia breathing (FiO_2_ = 12.5%) suggesting augmented RV function (Kjaergaard et al., 2007). Significantly decreased TAPSE values upon hypoxia breathing (FiO_2_ = 10%), suggesting RV systolic dysfunction, were observed in one study (Reichenberger et al., 2007). One more study evaluating TAPSE at several time points over the course of 150 min hypoxia showed that TAPSE decreased after 1 h of hypoxia breathing (Netzer et al., 2017).

Other frequently reported RV systolic function parameters included RV-MPI (Kjaergaard et al., 2007; Reichenberger et al., 2007; Hanaoka et al., 2011; Boos et al., 2013; Netzer et al., 2017; Seccombe et al., 2017) and RV-S′ (Huez et al., 2005; Kjaergaard et al., 2007; Pavelescu and Naeije, 2012; Boos et al., 2013; Netzer et al., 2017; Pezzuto et al., 2018), which were evaluated in 6 studies each. RV-MPI was not changed in 3 studies (Kjaergaard et al., 2007; Boos et al., 2013; Seccombe et al., 2017), while its values were significantly increased in one study (Reichenberger et al., 2007). In addition, RV-MPI values increased in subjects susceptible to HAPE (Hanaoka et al., 2011) and in those who were exposed to hypoxia for more than 100 min (Netzer et al., 2017). RV-S′ values remained unchanged in 4 studies (Huez et al., 2005; Kjaergaard et al., 2007; Pavelescu and Naeije, 2012; Pezzuto et al., 2018) and were increased in one study (Boos et al., 2013) or decreased in another study after 1 h hypoxia exposure (Netzer et al., 2017). RV-GLS was evaluated in 2 studies and showed opposite results (Goebel et al., 2013; Ewalts et al., 2021).

#### Right Ventricular Diastolic Function

In 6 studies, at least one of the following RV diastolic function parameters was reported: early diastolic tricuspid inflow velocity (RV-E), late diastolic tricuspid inflow velocity (RV-A), early-to-late diastolic tricuspid inflow velocities ratio (RV-E/A), tissue Doppler-derived early diastolic tricuspid annular velocity (RV-E′), tissue Doppler-derived late diastolic tricuspid annular velocity (RV-A′), tissue Doppler-derived early-to-late diastolic tricuspid annular velocities ratio (RV-E′/A′) (Huez et al., 2005; Kjaergaard et al., 2007; Pavelescu and Naeije, 2012; Boos et al., 2013; Goebel et al., 2013; Netzer et al., 2017). The RV diastolic parameters RV-E′ and RV-A′ were assessed in 5 (Huez et al., 2005; Kjaergaard et al., 2007; Pavelescu and Naeije, 2012; Boos et al., 2013; Netzer et al., 2017) and 4 studies (Huez et al., 2005; Kjaergaard et al., 2007; Pavelescu and Naeije, 2012; Netzer et al., 2017), respectively. In all these studies, RV-E′ remained unchanged upon exposure to acute hypoxia. In 3 studies, RV-A′ values increased upon hypoxia exposure suggesting increased right atrial (RA) contribution to the RV filling (Huez et al., 2005; Pavelescu and Naeije, 2012; Boos et al., 2013). Consequently, two studies reported decreased RV-E′/A′ values upon acute hypoxia exposure (Huez et al., 2005; Pavelescu and Naeije, 2012).

#### Right Ventricular Morphology

Seven of the studies reported at least one of the following RV morphology parameters: RV end diastolic area (RV-EDA), RV end systolic area (RV-ESA), RV basal diameter (RVD1), RV mid diameter (RVD2), RV longitudinal size (RVD3) (Huez et al., 2005; Kjaergaard et al., 2007; Pavelescu and Naeije, 2012; Netzer et al., 2017; Seccombe et al., 2017; Pezzuto et al., 2018; Ewalts et al., 2021). The most frequently evaluated RV morphology parameter RV-EDA was reported in 7 studies (Huez et al., 2005; Kjaergaard et al., 2007; Pavelescu and Naeije, 2012; Netzer et al., 2017; Seccombe et al., 2017; Pezzuto et al., 2018; Ewalts et al., 2021). None of the studies revealed changes in the RV morphology following simulated altitude exposure (Huez et al., 2005; Kjaergaard et al., 2007; Pavelescu and Naeije, 2012; Seccombe et al., 2017; Pezzuto et al., 2018; Ewalts et al., 2021), except one study, which showed RV dilation after 10% oxygen breathing (Netzer et al., 2017).

### Effects of High-Altitude Exposure on the Right Ventricle

Twenty-five studies were conducted in high-altitude field conditions and included 1144 healthy adults exposed to altitudes of 3100 to 7042 m (Huez et al., 2009; de Vries et al., 2010; Pratali et al., 2010; Page et al., 2013; Boos et al., 2014; Stembridge et al., 2014, 2019; Dedobbeleer et al., 2015; Hilty et al., 2016, 2019; Maufrais et al., 2017, 2019; Qiu et al., 2017; Berger et al., 2018; De Boeck et al., 2018; Sareban et al., 2020; Stewart et al., 2020; Tian et al., 2020; Yang et al., 2020a,b; Ewalts et al., 2021; Gaur et al., 2021; He et al., 2021; Lichtblau et al., 2021; Yuan et al., 2021). At high-altitude, echocardiographic examinations were conducted at various time points of the sojourn ranging from a few hours to 26 days. In some studies, serial echocardiographic investigations were performed over the course of several days of high-altitude stay (Huez et al., 2009; Page et al., 2013; Boos et al., 2014; Hilty et al., 2016, 2019; Maufrais et al., 2017; Berger et al., 2018; De Boeck et al., 2018; Sareban et al., 2020; Stewart et al., 2020; Gaur et al., 2021). In 3 of them, measurements were taken serially over a progressive altitude increment (Huez et al., 2009; Boos et al., 2014; Stewart et al., 2020). In 12 studies, both male and female subjects were included (Huez et al., 2009; de Vries et al., 2010; Pratali et al., 2010; Page et al., 2013; Boos et al., 2014; Dedobbeleer et al., 2015; Berger et al., 2018; De Boeck et al., 2018; Hilty et al., 2019; Stewart et al., 2020; Lichtblau et al., 2021; Yuan et al., 2021), while in 13 studies participants consisted of male subjects only (Stembridge et al., 2014, 2019; Hilty et al., 2016; Maufrais et al., 2017, 2019; Qiu et al., 2017; Sareban et al., 2020; Tian et al., 2020; Yang et al., 2020a,b; Ewalts et al., 2021; Gaur et al., 2021; He et al., 2021). Mode of ascent also differed significantly between the studies ranging from a rapid transportation to high-altitude by airplanes to a staged acclimatization with spending nights at intermediate altitudes. Echocardiographic examination included assessment of RV systolic and diastolic function and morphology. The summary of the assessed RV parameters is presented in Table 5 and the changes of the parameters following high-altitude exposure are summarized in Table 6.

**Table 5:** Echocardiographic parameters assessed in acute high-altitude exposure studies.

**Studies**	**Diastolic function**						**Systolic function**					**Morphology**				
	RV-E	RV-A	RV-E/A	RV-E′	RV-A′	RV-E′/A′	RV-S′	TAPSE	RV-FAC	RV-MPI	RV-GLS	RVD1	RVD2	RVD3	RV-EDA	RV-ESA
Huez et al., 2009	✓	✓		✓	✓	✓	✓	✓		✓						
de Vries et al., 2010				✓				✓								
Pratali et al., 2010								✓								
Page et al., 2013			✓				✓	✓		✓	✓					
Boos et al., 2014	✓			✓			✓									
Stembridge et al., 2014				✓	✓		✓	✓			✓				✓	✓
Dedobbeleer et al., 2015							✓	✓	✓	✓						
Hilty et al., 2016								✓	✓	✓					✓	✓
De Boeck et al., 2018							✓	✓							✓	✓
Qiu et al., 2017										✓						
Maufrais et al., 2017	✓	✓	✓	✓	✓		✓	✓	✓		✓				✓	✓
Berger et al., 2018							✓	✓	✓	✓						
Hilty et al., 2019								✓								
Stembridge et al., 2019	✓	✓	✓						✓		✓				✓	✓
Maufrais et al., 2019	✓	✓	✓	✓	✓		✓	✓	✓							
Sareban et al., 2020							✓	✓	✓	✓	✓	✓	✓	✓	✓	✓
Stewart et al., 2020				✓	✓	✓	✓	✓	✓						✓	✓
Yang et al., 2020b			✓	✓			✓		✓						✓	✓
Yang et al., 2020a			✓	✓			✓	✓	✓		✓				✓	
Tian et al., 2020	✓	✓	✓	✓			✓		✓	✓					✓	✓
Ewalts et al., 2021									✓		✓				✓	✓
Gaur et al., 2021	✓	✓	✓	✓	✓	✓	✓	✓		✓		✓	✓	✓		
Yuan et al., 2021	✓	✓	✓	✓			✓	✓	✓		✓	✓	✓		✓	✓
He et al., 2021	✓	✓	✓	✓	✓	✓	✓		✓						✓	✓
Lichtblau et al., 2021								✓	✓							

*✓Indicates that the echocardiographic parameter was provided in the corresponding studies; empty cell indicate that the echocardiographic parameter is not provided in the corresponding studies*.

**Table 6:** Summary of the studies evaluating the impact of high-altitude exposure on the right ventricle.

**Study**	**Measurement timepoints (hours or days)**	**Altitude (m)**	**Systolic function parameters**	**Diastolic function parameters**	**Morphology parameters**
Huez et al., 2009	1 day	3,750	↔ RV-S′, ↔ TAPSE, ↑ RV-MPI	↓ RV-E, ↔ RV-A, ↓ RV-E/A, ↔ RV-E′, ↔ RV-A′, ↔ RV-E′/A′	
	10 days	4,850	↔ RV-S′, ↔TAPSE, ↑ RV-MPI	↓ RV-E, ↔ RV-A, ↓ RV-E/A, ↔ RV-E′, ↔ RV-A′, ↔ RV-E′/A′	
de Vries et al., 2010	10 days	4,200	↓ TAPSE	↔RV-E′	
Pratali et al., 2010	6 days	3,440	↔ TAPSE		
	15 days	5,130	↔ TAPSE		
Page et al., 2013	4 days	3,450	↔ RV-S′, ↔ TAPSE, ↑ RV-MPI, ↔ RV-GLS	↓ RV-E/A	
	7 days	4,730	↔ RV-S′, ↔ TAPSE, ↑ RV-MPI, ↔ RV-GLS	↓ RV-E/A	
Boos et al., 2014	2 days	3,440	↔ RV-S′	↔ RV-E, ↔RV-E′	
	6 days	4,270	↔ RV-S′	↔ RV-E, ↔RV-E′	
	9 days	5,150	↔ RV-S′	↔ RV-E, ↔RV-E′	
Stembridge et al., 2014	10 days	5,050	↓ TAPSE, ↔ RV-S′, ↓ RV-GLS	↔ RV-E′, ↔ RV-A′	↔ RV-EDA, ↔ RV-ESA
Dedobbeleer et al., 2015	4 days	4,350	↔ RV-S′, ↓ TAPSE, ↑ RV-MPI, ↔ RV-FAC		
Hilty et al., 2016	2–3 days	3,454	↔ TAPSE, ↔ RV-FAC, ↑ RV-MPI		↔ RV-EDA
	8–10 days	3,454	↔ TAPSE, ↔ RV-FAC, ↑ RV-MPI		↔ RV-EDA
	17–18 days	3,454	↔ TAPSE, ↔ RV-FAC, ↑ RV-MPI		↔ RV-EDA
	26–27 days	3,454	↔ TAPSE, ↔ RV-FAC, ↑ RV-MPI		↔ RV-EDA
De Boeck et al., 2018	3 days	4,559	↔ RV-FAC, ↔ RV-S′, ↔ TAPSE		↑ RV-EDA, ↑ RV-ESA
	4 days	4,559	↔ RV-FAC, ↑ RV-S′, ↔ TAPSE		↔ RV-EDA, ↔ RV-ESA
Qiu et al., 2017	1 day	3,700	↑ RV-MPI		
Maufrais et al., 2017	0.5 days	4,350	↔ RV-FAC, ↔ TAPSE, ↔ RV-S′, ↔ RV-GLS	↔ RV-E, ↑RV-A, ↓ RV-E/A, ↑ RV-E′, ↑ RV-A′	↔ RV-EDA, ↔ RV-ESA
	2 days	4,350	↔ RV-FAC, ↔ TAPSE, ↔ RV-S′, ↔ RV-GLS	↔ RV-E, ↔RV-A, ↓ RV-E/A, ↔RV-E′, ↑ RV-A′	↔ RV-EDA, ↔ RV-ESA
	4 days	4,350	↔ RV-FAC, ↔ TAPSE, ↔ RV-S′, ↔ RV-GLS	↔ RV-E, ↔RV-A, ↓ RV-E/A, ↔RV-E′, ↔RV-A′,	↔ RV-EDA, ↔ RV-ESA
	6 days	4,350	↔ RV-FAC, ↔ TAPSE, ↔ RV-S′, ↔ RV-GLS	↔ RV-E, ↔RV-A, ↓ RV-E/A, ↔RV-E′, ↔RV-A′	↔ RV-EDA, ↔ RV-ESA
Berger et al., 2018	7 h	4,559	↑ TAPSE, ↔ RV-MPI, ↑ RV-S′, ↔ RV-FAC		
	20 h	4,559	↑ TAPSE, ↔ RV-MPI ↑ RV-S′, ↔ RV-FAC		
	44 h	4,559	↑ TAPSE, ↔ RV-MPI ↑ RV-S′, ↔ RV-FAC		
Stembridge et al., 2019	5–10 days	3,800	↔ RV-FAC, ↔ RV-GLS	↔ RV-E, ↔RV-A, ↔ RV-E/A	↔ RV-EDA, ↔ RV-ESA
Maufrais et al., 2019	10 days	5,058	↔ RV-FAC, ↔ TAPSE, ↑ RV-S′, ↔ RV-GLS	↔ RV-E, ↑ RV-A, ↓ RV-E/A, ↔ RV-A′, ↔RV-E′,↔ RV-E′/A′	
Hilty et al., 2019	8 days	6,022	↓ TAPSE		
	15 days	7,042	↔ TAPSE		
Ewalts et al., 2021	5–10 days	3,800	↔ RV-FAC, ↔ RV-SV, ↔ RV-GLS		↔ RV-EDA, ↔ RV-ESA
Tian et al., 2020	5 days	4,100	↓ RV-FAC, ↔ RV-S′, ↑ RV-MPI	↓ RV-E, ↓ RV-A, ↓ RV-E/A, ↔ RV-E′	↓ RV-EDAI, ↔ RV-ESAI
Yang et al., 2020b	1 day	4,100	↓ RV-FAC, ↔ RV-S′,	↓ RV-E/A, ↔RV-E′	↔ RV-EDA, ↔ RV-ESA
Yang et al., 2020a	1 day	4,100	↓ RV-FAC, ↓ TAPSE, ↔ RV-S′, ↓ RV-GLS,	↓ RV-E/A, ↔RV-E′,	↓ RV-EDA, ↔ RV-ESA
(Young) (Stewart et al., 2020)	3 days	3,100	↓ RV-FAC, ↔ RV-S′, ↓TAPSE	↔ RV-A′, ↔RV-E′, ↔ RV-E′/A′	↑ RV-EDA, ↑ RV-ESA
	8 days	4,800	↓ RV-FAC, ↑ RV-S′, ↓TAPSE	↑ RV-A′, ↔RV-E′, ↓ RV-E′/A′	↑ RV-EDA, ↑ RV-ESA
(Old) (Stewart et al., 2020)	3 days	3,00	↓ RV-FAC, ↑ RV-S′, ↓TAPSE	↑ RV-A′, ↔RV-E′, ↓ RV-E′/A′	↑ RV-EDA, ↑ RV-ESA
	8 days	4,800	↓ RV-FAC, ↑ RV-S′, ↓TAPSE	↑ RV-A′, ↔RV-E′, ↓ RV-E′/A′	↑ RV-EDA, ↑ RV-ESA
Sareban et al., 2020	7 h	4,559	↔ TAPSE, ↔ RV-FAC, ↑ RV-S′, ↔ RV-MPI, ↔ RV-GLS		↔ RVD1, ↔ RVD2, ↔ RVD3, ↔ RV-EDA, ↔ RV-ESA
	20 h	4,559	↑ TAPSE, ↔ RV-FAC, ↑ RV-S′, ↔ RV-MPI, ↔ RV-GLS		↔ RVD1, ↔ RVD2, ↔ RVD3, ↔ RV-EDA, ↔ RV-ESA
	44 h	4,559	↔ TAPSE, ↔ RV-FAC, ↑ RV-S′, ↔ RV-MPI, ↔ RV-GLS		↔ RVD1, ↔ RVD2, ↔ RVD3, ↔ RV-EDA, ↔ RV-ESA
(Kyrgyz) (Gaur et al., 2021)	3 days	4,111	↔ TAPSE, ↔ RV-S′, ↔ RV-MPI	↔ RV-A, ↔ RV-E, ↔ RV-E/A, ↔ RV-E′, ↔ RV-A′, ↔ RV-E′/A′	↔ RVD1, ↔ RVD2, ↔ RVD3
	7 days	4,111	↔ TAPSE, ↔ RV-S′, ↔ RV-MPI	↔ RV-A, ↔ RV-E, ↔ RV-E/A, ↔ RV-E′, ↔ RV-A′, ↔ RV-E′/A′	↔ RVD1, ↔ RVD2, ↔ RVD3
	14 days	411	↔ TAPSE, ↔ RV-S′, ↔ RV-MPI	↔ RV-A, ↔ RV-E, ↔ RV-E/A, ↔ RV-E′, ↓ RV-A′, ↔ RV-E′/A′	↔ RVD1, ↔ RVD2, ↔ RVD3
	21 days	4,111	↓ TAPSE, ↔ RV-S′, ↔ RV-MPI	↔ RV-A, ↔ RV-E, ↔ RV-E/A, ↓ RV-E′, ↓ RV-A′, ↔ RV-E′/A′	↔ RVD1, ↔ RVD2, ↔ RVD3
(Indians) (Gaur et al., 2021)	3 days	4,111	↔ TAPSE, ↔ RV-S′, ↔ RV-MPI	↔ RV-A, ↔ RV-E, ↔ RV-E/A, ↔ RV-E′, ↔ RV-A′, ↔ RV-E′/A′	↔ RVD1, ↔ RVD2, ↔ RVD3
	7 days	4,111	↔ TAPSE, ↔ RV-S′, ↔ RV-MPI	↔ RV-A, ↔ RV-E, ↔ RV-E/A, ↓ RV-E′, ↔ RV-A′, ↔ RV-E′/A′	↔ RVD1, ↔ RVD2, ↔ RVD3
	14 days	4,111	↓ TAPSE, ↔ RV-S′, ↔ RV-MPI	↔ RV-A, ↓ RV-E, ↔ RV-E/A, ↓ RV-E′,↔ RV-A′, ↔ RV-E′/A′	↔ RVD1, ↔ RVD2, ↔ RVD3
	21 days	4,111	↔ TAPSE, ↔ RV-S′, ↔ RV-MPI	↔ RV-A, ↔ RV-E, ↔ RV-E/A, ↓ RV-E′, ↔ RV-A′, ↔ RV-E′/A′	↔ RVD1, ↔ RVD2, ↔ RVD3
He et al., 2021	5–2 h	4,100	↓ RV-FAC, ↔ RV-S′	↓ RV-E, ↓ RV-A, ↓ RV-E/A, ↔RV-E′, ↓ RV-A′	↓ RV-EDAI, ↔ RV-ESAI
Lichtblau et al., 2021	4–8 h	5,050	↔ TAPSE, ↔ RV-FAC		
Yuan et al., 2021	15 ± 3 h	4,100	↓ TAPSE, ↓ RV-FAC, ↑ RV-S′, ↓ RV-GLS	↓ RV-E, ↑ RV-A, ↓ RV-E/A, ↔ RV-E′	↔ RVD1, ↑ RVD2, ↔ RV-EDA, ↔ RV-EDA

*↑, high-altitude values are significantly increased compared to baseline values; ↓, high-altitude values are significantly decreased compared to baseline values; ↔ high-altitude values are not changed significantly compared to baseline values*.

#### Right Ventricular Systolic Function

In all studies, at least one of the following RV systolic function parameters was reported: TAPSE, RV-S′, RV-FAC or RV-MPI. In addition, RV free wall or global strain were assessed in 8 studies (Page et al., 2013; Stembridge et al., 2014, 2019; Maufrais et al., 2017; Sareban et al., 2020; Yang et al., 2020a,b; Ewalts et al., 2021). The most frequently reported RV systolic function parameter TAPSE was assessed in 18 studies (Huez et al., 2009; de Vries et al., 2010; Pratali et al., 2010; Page et al., 2013; Stembridge et al., 2014; Dedobbeleer et al., 2015; Hilty et al., 2016, 2019; Maufrais et al., 2017, 2019; Berger et al., 2018; De Boeck et al., 2018; Sareban et al., 2020; Stewart et al., 2020; Yang et al., 2020a; Gaur et al., 2021; Lichtblau et al., 2021; Yuan et al., 2021). In 6 of these studies (de Vries et al., 2010; Stembridge et al., 2014; Dedobbeleer et al., 2015; Maufrais et al., 2019; Lichtblau et al., 2021; Yuan et al., 2021), TAPSE was evaluated once while in others it was measured at two (Huez et al., 2009; Pratali et al., 2010; Page et al., 2013; De Boeck et al., 2018; Hilty et al., 2019; Stewart et al., 2020), or 3 (Berger et al., 2018; Sareban et al., 2020) or 4 (Hilty et al., 2016; Maufrais et al., 2017; Sareban et al., 2020; Gaur et al., 2021) time-points over the course of high altitude acclimatization. Regardless of measured time-points in 8 studies out of 18, TAPSE values were maintained during high-altitude stay suggesting preserved RV systolic function (Huez et al., 2009; Pratali et al., 2010; Page et al., 2013; Hilty et al., 2016; Maufrais et al., 2017, 2019; De Boeck et al., 2018; Lichtblau et al., 2021). Only one study reported increased TAPSE values upon high-altitude exposure (Berger et al., 2018). However, other six studies reported reduced TAPSE values at high-altitude on days 3, 4, 8, 10, 12, and 14 suggesting RV systolic dysfunction (de Vries et al., 2010; Stembridge et al., 2014; Dedobbeleer et al., 2015; Stewart et al., 2020; Yang et al., 2020a; Yuan et al., 2021). In addition, three studies reported inconsistent changes in TAPSE values over the course of high-altitude acclimatization (Hilty et al., 2019; Sareban et al., 2020; Gaur et al., 2021).

Another frequently evaluated RV systolic function parameter RV-S′ was reported in 17 studies (Huez et al., 2009; Page et al., 2013; Boos et al., 2014; Stembridge et al., 2014; Dedobbeleer et al., 2015; Maufrais et al., 2017, 2019; Berger et al., 2018; De Boeck et al., 2018; Sareban et al., 2020; Stewart et al., 2020; Tian et al., 2020; Yang et al., 2020a,b; Gaur et al., 2021; He et al., 2021; Yuan et al., 2021). In 11 studies, RV-S′ values were maintained up to 21 days of high-altitude sojourn suggesting preserved RV systolic function (Huez et al., 2009; Page et al., 2013; Boos et al., 2014; Stembridge et al., 2014; Dedobbeleer et al., 2015; Maufrais et al., 2017; Tian et al., 2020; Yang et al., 2020a,b; Gaur et al., 2021; He et al., 2021). Moreover, in 5 studies, RV-S′ values were significantly increased on days 3, 8, and 10 of high-altitude stay, suggesting enhanced RV performance (Berger et al., 2018; De Boeck et al., 2018; Sareban et al., 2020; Stewart et al., 2020; Yang et al., 2020a; Yuan et al., 2021).

Similarly, no changes in RV-FAC were revealed in 9 studies out of 15 (Dedobbeleer et al., 2015; Hilty et al., 2016; Maufrais et al., 2017; Berger et al., 2018; De Boeck et al., 2018; Stembridge et al., 2019; Sareban et al., 2020; Ewalts et al., 2021; Lichtblau et al., 2021). In contrast, in other 6 studies, decreased RV-FAC upon acute high-altitude induction suggested reduced RV systolic function (Stewart et al., 2020; Tian et al., 2020; Yang et al., 2020a,b; He et al., 2021; Yuan et al., 2021). Maintained RV-MPI over the course of high-altitude stay was reported in 3 studies out of 9 (Berger et al., 2018; Sareban et al., 2020; Gaur et al., 2021). In other 6 studies, elevated RV-MPI values suggested compromised RV function (Huez et al., 2009; Page et al., 2013; Dedobbeleer et al., 2015; Hilty et al., 2016; Qiu et al., 2017; Tian et al., 2020). Unchanged RV-GLS was revealed in 6 studies out of 8 suggesting preserved RV function (Page et al., 2013; Maufrais et al., 2017, 2019; Stembridge et al., 2019; Sareban et al., 2020; Ewalts et al., 2021). Reduced RV-GLS values were reported in other 3 studies (Stembridge et al., 2014; Yang et al., 2020a; Yuan et al., 2021).

#### Right Ventricular Diastolic Function

Fifteen studies reported at least one of the following RV diastolic function parameters upon high-altitude exposure: RV-E, RV-A, RV-E/A, RV-E′, RV-A′, or RV-E′/A′(Huez et al., 2009; de Vries et al., 2010; Page et al., 2013; Boos et al., 2014; Stembridge et al., 2014, 2019; Maufrais et al., 2017, 2019; Stewart et al., 2020; Tian et al., 2020; Yang et al., 2020a,b; Gaur et al., 2021; He et al., 2021; Yuan et al., 2021). The most frequently evaluated parameter was RV-E′ (Huez et al., 2009; de Vries et al., 2010; Boos et al., 2014; Stembridge et al., 2014; Maufrais et al., 2017, 2019; Stewart et al., 2020; Tian et al., 2020; Yang et al., 2020a,b; Gaur et al., 2021; He et al., 2021; Yuan et al., 2021). In 11 studies out of 13, no changes in RV-E′ values were revealed at various time-points and altitude levels (Huez et al., 2009; de Vries et al., 2010; Boos et al., 2014; Stembridge et al., 2014; Maufrais et al., 2019; Stewart et al., 2020; Tian et al., 2020; Yang et al., 2020a,b; He et al., 2021; Yuan et al., 2021). In one study, RV-E′ values increased at an early time point of high-altitude acclimatization and subsequently returned to baseline values at later stages (Maufrais et al., 2017), while they decreased after 3 weeks of high-altitude stay in another study (Gaur et al., 2021).

Another frequently evaluated diastolic parameter RV-E/A was reported in 11 studies (Huez et al., 2009; Page et al., 2013; Maufrais et al., 2017, 2019; Stembridge et al., 2019; Tian et al., 2020; Yang et al., 2020a,b; Gaur et al., 2021; He et al., 2021; Yuan et al., 2021). RV-E/A was decreased in 9 studies (Huez et al., 2009; Page et al., 2013; Maufrais et al., 2017, 2019; Tian et al., 2020; Yang et al., 2020a,b; He et al., 2021; Yuan et al., 2021) and it was unchanged in two studies (Stembridge et al., 2019; Gaur et al., 2021). In 5 studies out of 9, RV-E values remained unaffected at high-altitude (Boos et al., 2014; Maufrais et al., 2017, 2019; Stembridge et al., 2019; Gaur et al., 2021), and they were significantly reduced in 4 studies (Huez et al., 2009; Tian et al., 2020; He et al., 2021; Yuan et al., 2021). RV-A values were not affected upon high altitude exposure in 3 studies out of 8 (Huez et al., 2009; Stembridge et al., 2019; Gaur et al., 2021) and were reduced in 2 studies (Tian et al., 2020; He et al., 2021). Three studies reported significantly increased RV-A values at high-altitude (Maufrais et al., 2017, 2019; Yuan et al., 2021). RV-E′/A′ was reported in 4 studies, which showed preserved values in 2 studies (Huez et al., 2009; Maufrais et al., 2019) and reduced values in one study (Stewart et al., 2020; Gaur et al., 2021).

#### Right Ventricular Morphology

Fourteen studies reported at least one of the following RV morphology parameters: RV-EDA, RV-ESA, RVD1, RVD2, or RVD3 (Stembridge et al., 2014, 2019; Hilty et al., 2016; Maufrais et al., 2017; De Boeck et al., 2018; Sareban et al., 2020; Stewart et al., 2020; Tian et al., 2020; Yang et al., 2020a,b; Ewalts et al., 2021; Gaur et al., 2021; He et al., 2021; Yuan et al., 2021). The most frequently reported RV morphology parameters were RV-EDA and RV-ESA (Stembridge et al., 2014, 2019; Hilty et al., 2016; Maufrais et al., 2017; De Boeck et al., 2018; Sareban et al., 2020; Stewart et al., 2020; Tian et al., 2020; Yang et al., 2020a,b; Ewalts et al., 2021; He et al., 2021; Yuan et al., 2021). In some studies, RV-EDA and RV-ESA values were indexed to body surface area (Tian et al., 2020; He et al., 2021). In 8 studies out of 13, RV-EDA values remained unchanged upon high-altitude exposure (Stembridge et al., 2014, 2019; Hilty et al., 2016; Maufrais et al., 2017; Sareban et al., 2020; Yang et al., 2020b; Ewalts et al., 2021; Yuan et al., 2021), while they were increased in 2 (De Boeck et al., 2018; Stewart et al., 2020) and decreased in 3 studies (Tian et al., 2020; Yang et al., 2020a; He et al., 2021). Similarly, high-altitude induction did not alter RV-ESA values in 10 studies (Stembridge et al., 2014, 2019; Maufrais et al., 2017; Sareban et al., 2020; Tian et al., 2020; Yang et al., 2020a,b; Ewalts et al., 2021; He et al., 2021; Yuan et al., 2021) and was associated with increased RV-ESA values in 2 studies (De Boeck et al., 2018; Stewart et al., 2020).

Three studies reported unaltered RVD1 and RVD2 values upon high-altitude exposure (Sareban et al., 2020; Gaur et al., 2021; Yuan et al., 2021). Similarly, RVD3 values remained unchanged at high-altitude in 2 studies (Sareban et al., 2020; Gaur et al., 2021).

## Discussion

The present systematic review aimed to analyze the effects of acute altitude simulation and high-altitude exposure on the echocardiography-based RV systolic and diastolic function as well as RV morphology parameters in healthy lowland individuals. Both, simulated altitude and high-altitude field conditions have been used to study the effects of reduced arterial partial oxygen pressure on the RV function and morphology. Literature search identified 37 eligible articles that assessed at least one of the RV echocardiography parameters at sea level and at various time points after hypoxia exposure. Of these, 12 studies were conducted using simulated altitude (Huez et al., 2005; Kjaergaard et al., 2007; Reichenberger et al., 2007; Hanaoka et al., 2011; Pavelescu and Naeije, 2012; Boos et al., 2013, 2016; Goebel et al., 2013; Netzer et al., 2017; Seccombe et al., 2017; Pezzuto et al., 2018; Ewalts et al., 2021) and 25 studies were performed in high-altitude field conditions (Huez et al., 2009; de Vries et al., 2010; Pratali et al., 2010; Page et al., 2013; Boos et al., 2014; Stembridge et al., 2014, 2019; Dedobbeleer et al., 2015; Hilty et al., 2016, 2019; Maufrais et al., 2017, 2019; Qiu et al., 2017; Berger et al., 2018; De Boeck et al., 2018; Sareban et al., 2020; Stewart et al., 2020; Tian et al., 2020; Yang et al., 2020a,b; Ewalts et al., 2021; Gaur et al., 2021; He et al., 2021; Lichtblau et al., 2021; Yuan et al., 2021). The simulated altitude studies have exploited either hypoxic breathing (Huez et al., 2005; Kjaergaard et al., 2007; Hanaoka et al., 2011; Pezzuto et al., 2018; Ewalts et al., 2021) or normobaric hypoxia (Goebel et al., 2013; Boos et al., 2016; Netzer et al., 2017) or hypobaric hypoxia chambers (Boos et al., 2013; Goebel et al., 2013).

### Right Ventricular Systolic Function

Only a few studies followed the international guidelines and assessed multiple parameters for the comprehensive RV evaluation (Pavelescu and Naeije, 2012; Boos et al., 2013; Maufrais et al., 2017; Netzer et al., 2017; Stewart et al., 2020; Tian et al., 2020; Yang et al., 2020a; Gaur et al., 2021; Yuan et al., 2021). Most of the studies have evaluated only one or 2 or 3 of the following RV systolic function parameters: TAPSE, RV-S′, RV-FAC, RV-MPI or RV-GLS.

Assessment of multiple parameters revealed inconsistent changes in RV systolic function parameters at different time-points and altitude levels. More specifically, TAPSE and RV-S′ remained unchanged upon exposure to high-altitude in the majority of the studies (Huez et al., 2009; Page et al., 2013; Hilty et al., 2016; Maufrais et al., 2017; Gaur et al., 2021). However, in a few studies, RV function assessed by TAPSE was augmented at earlier time-points of exposure (with 24 h) (Berger et al., 2018; Sareban et al., 2020) and then decreased at later stages of high-altitude stay (Stewart et al., 2020; Gaur et al., 2021). Similarly, RV-S′ values were increased at certain time-points of high-altitude exposure in several studies (Berger et al., 2018; De Boeck et al., 2018; Sareban et al., 2020; Stewart et al., 2020; Yang et al., 2020a). Notably, assessment of RV-FAC and RV-MPI revealed augmented RV systolic function in none of the studies (Huez et al., 2009; Page et al., 2013; Dedobbeleer et al., 2015; Maufrais et al., 2017; Berger et al., 2018; De Boeck et al., 2018; Stembridge et al., 2019; Sareban et al., 2020; Stewart et al., 2020; Tian et al., 2020; Yang et al., 2020a; Ewalts et al., 2021; He et al., 2021; Lichtblau et al., 2021; Yuan et al., 2021). In fact, in several studies, the changes of RV-FAC and RV-MPI values indicated diminished RV systolic function (Huez et al., 2009; Page et al., 2013; Dedobbeleer et al., 2015; Hilty et al., 2016; Qiu et al., 2017; Stewart et al., 2020; Tian et al., 2020; Yang et al., 2020a; He et al., 2021; Yuan et al., 2021). Moreover, serial assessment of the RV systolic function parameters within the same study showed inconsistent changes over the course of high-altitude acclimatization (Sareban et al., 2020; Stewart et al., 2020; Gaur et al., 2021). Taken together, in the majority of the studies conventional echocardiography demonstrated maintained RV systolic function upon acute high-altitude exposure, although with variations in the directions of changes in RV systolic parameters and measurement points over the course of high-altitude acclimatization.

Recently, the advent of speckle-tracking echocardiography and its utilization to assess myocardial strain allowed less angle-dependent and load-independent insights into intrinsic myocardial function (Salvo et al., 2015). Speckle-tracking echocardiography has been shown to be a reliable technique in the analysis of RV myocardial mechanics (Kossaify, 2015). In addition, it enables assessment of additional alterations of myocardial mechanics such as contraction dyssynchrony (Badagliacca et al., 2015). Using this technique, RV dyssynchrony was shown in various cardiopulmonary conditions associated with an increased RV pressure overload (Kalogeropoulos et al., 2008; Badagliacca et al., 2017; Pezzuto et al., 2018). Speckle-tracking echocardiography was utilized to evaluate RV-GLS in two simulated altitude studies (Goebel et al., 2013; Ewalts et al., 2021) and in several high-altitude studies (Page et al., 2013; Maufrais et al., 2017; Stembridge et al., 2019; Sareban et al., 2020; Yang et al., 2020a; Ewalts et al., 2021; Yuan et al., 2021). Although, the RV-GLS values remained unchanged upon hypoxia exposure in most of the studies (Page et al., 2013; Maufrais et al., 2017, 2019; Stembridge et al., 2019; Sareban et al., 2020; Ewalts et al., 2021), three studies demonstrated RV dyssynchrony in healthy adults upon exposure to acute simulated altitude and high-altitude (Pezzuto et al., 2018; Ewalts et al., 2021; Yuan et al., 2021).

Augmented RV afterload predisposes the RV to myocardial dysfunction. Although, there is significant amount of information on the pressure overload-induced RV dysfunction, hypoxia itself induces activation of several molecular pathways that could contribute to RV remodeling and dysfunction (Pena et al., 2020). In addition, preclinical studies have demonstrated that hypoxia can directly exert negative inotropic effects in intact animal preparations (Tucker et al., 1976) and in isolated cardiomyocytes (Silverman et al., 1997). The depressive effects of hypoxia on embryonic mouse cardiomyocytes may be related to hypoxia-inducible factor-1 mediated sarcoplasmic reticulum Ca^2+^-ATPase downregulation (Ronkainen et al., 2011). Direct negative inotropic effects of hypoxia were corroborated in a recent study that demonstrated preservation of the RV contractile function by improved oxygen delivery to the hypoxic myocardium in lambs exposed to acute hypoxia (Boehme et al., 2018).

Besides the direct effects of hypoxia on the myocardium, reduced venous return due to plasma volume contraction might also be responsible for the RV dyssynchrony as increasing venous return to the right heart alleviates it (Ewalts et al., 2021). In addition, in a recent study, high-altitude-induced RV dyssynchrony was associated with a lower degree of oxygen saturation and higher PAP (Yang et al., 2020a) suggesting its relationship with hypoxemia and increased RV afterload. Interestingly, there were no differences in the conventional echocardiography systolic function parameters between subjects with RV dyssynchrony and those without (Yang et al., 2020a). Taken together, while conventional echocardiography-derived parameters suggest maintained RV systolic function upon exposure to hypoxia, subtle changes can be revealed by speckle-tracking echocardiography. Interestingly, the findings derived by the latter suggested that these changes might be driven not only by increased afterload but also by other factors such as decreased preload and direct effects of hypoxia.

High-altitude exposure causes major changes in various functions of the cardiovascular system (Naeije, 2010). In addition to HPV and an associated increase in PAP and PVR, other initial cardiovascular responses include an increase in cardiac output along with tachycardia (Naeije, 2010). Of note, non-cardiovascular factors might contribute to the cardiovascular responses at high-altitude. For example, previous studies demonstrated plasma volume contraction upon high-altitude exposure (Singh et al., 1990), which can remain below sea level values even for up to 4–6 months above 4,000 m (Pugh, 1964). Reduced plasma volume causes, in turn, alterations in the LV function (Stembridge et al., 2019). These findings are supported by recent studies with plasma volume expansion (Stembridge et al., 2019). In healthy lowlanders, plasma volume expansion during acclimatization to 3,800 m restored LV end-diastolic volume, mitral inflow velocities and stroke volume (Stembridge et al., 2019).

Differences in ethnicity (Gaur et al., 2021), age (Stewart et al., 2020), gender (Boos et al., 2016) and exposure duration (Gaur et al., 2021) and physical activity might account for the discrepancies between the study results. The magnitude of the RV response to high-altitude exposure might also be related to the susceptibility to HAPE. For example, exposure to normobaric hypoxia was associated with RV dysfunction in Japanese HAPE-susceptible subjects, while HAPE-resistant subjects displayed maintained RV function (Hanaoka et al., 2011). In line with these findings, in healthy subjects at high-altitude, the degree of RV dysfunction was associated with subclinical pulmonary edema (Page et al., 2013). Importantly, HAPE-susceptible individuals exhibit an exaggerated HPV and display higher PAP values at high-altitude compared to HAPE-resistant subjects (Hanaoka et al., 2011). There is anecdotal evidence of severe acute hypoxic PH with RV dilatation in a previously healthy lowlander upon arrival to 3,700 m within 24 h (Huez et al., 2007). All these findings suggest that lowland apparently healthy HAPE-susceptible subjects are also prone to develop RV dysfunction, most probably due to an augmented HPV. However, direct effects of the gene-variants associated with HAPE susceptibility on the RV cannot be ruled out yet.

### Right Ventricular Diastolic Function

It should be noted that only a few studies followed the international guidelines to evaluate multiple parameters for full characterization of the RV diastolic function. Most of the studies provided only a few RV diastolic function parameters. Thus, 7 out of 12 simulated altitude and 15 out of 24 high-altitude exposure studies reported at least one of the following RV diastolic function parameters: RV-E, RV-A, RV-E/A, RV-E′, RV-A′, and RV-E′/A′.

The most consistent change upon high-altitude exposure was the decrease in RV-E/A (Huez et al., 2009; Page et al., 2013; Maufrais et al., 2017, 2019; Tian et al., 2020; Yang et al., 2020a; He et al., 2021; Yuan et al., 2021). However, in a few studies, RV-E/A values remained unchanged (Stembridge et al., 2019; Gaur et al., 2021). Interestingly, the values of RV-E and RV-A were unaffected in the majority of the studies (Boos et al., 2014; Stembridge et al., 2014, 2019; Maufrais et al., 2017, 2019; Gaur et al., 2021). In contrast to the conventional Doppler echocardiography, only a few studies reported changes of RV-E′, RV-A′, RV-E′/A′ in the TDI-derived RV filling parameters (Huez et al., 2009; de Vries et al., 2010; Boos et al., 2014; Stembridge et al., 2014; Maufrais et al., 2019; Stewart et al., 2020; Tian et al., 2020; Yang et al., 2020a; He et al., 2021; Yuan et al., 2021).

Hypoxia has been shown to alter myocardial relaxation (Tucker et al., 1976; Gibbs, 2007). However, LV and RV intrinsic relaxation estimated by TDI echocardiography derived E′ remained unchanged upon acute exposure to high-altitude suggesting that ventricular early filling may be unaffected by hypoxia (He et al., 2021). Interventricular interaction between the RV and LV may impair LV filling through the septal shift toward the LV due to RV pressure overload (B?rtsch and Gibbs, 2007). Alterations in the traditional LV diastolic function markers such as E/A ratio at high-altitude may not be associated with an impairment in myocardial relaxation but might rather reflect lower LV filling pressure (Holloway et al., 2011). Impairment of the LV relaxation seems less likely because pulmonary capillary wedge pressure, which is considered as a surrogate marker of left atrial pressure, is not elevated upon exposure to high-altitude (Reeves et al., 1987). Moreover, increased LV untwist velocity upon acute high-altitude exposure despite attenuated LV filling (Dedobbeleer et al., 2015; Stembridge et al., 2015; Osculati et al., 2016), suggests preserved or even augmented LV relaxation. Increased sympathetic activation may contribute to the enhanced LV untwisting (Rademakers et al., 1992). Likewise, changes in RV diastolic filling patterns upon hypoxia exposure may develop due to increased RV afterload and sympathetic nervous system activation but not due to altered RV relaxation (Huez et al., 2005, 2009; Naeije, 2010).

Atrial contraction is the final component of the ventricular diastole and contributes ~15–20% of stroke volume (He et al., 2021), and thus is intricately related to cardiac performance. As atria actively participate in the ventricular filling, atrial function is considered as a part of ventricular diastolic function. Atrial function can be affected by a number of factors such as decreased energy supply and preload, increased afterload, and altered ventricular mechanics observed upon exposure to high-altitude (Yang et al., 2020a; Ewalts et al., 2021; He et al., 2021). However, despite its importance, literature on atrial function at high-altitude remains scarce. Only a few studies evaluated atrial function upon exposure to high-altitude using speckle-tracking echocardiography (Sareban et al., 2020; He et al., 2021). Thus, unchanged left atrial and enhanced RA contractile function was reported a few hours following an ascent to 4,559 m (Sareban et al., 2019, 2020). In contrast, a more recent study revealed a slightly impaired left atrial function and a prominent RA dysfunction at high-altitude (He et al., 2021). More specifically, acute high-altitude exposure led to a decrease of the reservoir and conduit functions of both atria and reduced RA contractility (He et al., 2021). Notably, atrial dyssynchrony occurred only to the RA and was associated with worse RV performance (He et al., 2021). Taken together, in addition to conventional RV diastolic indices, evaluating RA function by means of speckle-tracking echocardiography can provide additional insights into the RV diastolic function in response to high-altitude exposure. However, exploitation of such methods remains underutilized.

### Clinical Implication of Acute Hypoxia Induced Right Ventricular Dysfunction

Understanding the physiology of the RV responses to acute hypoxia is important for gaining insights into mechanisms underlying acute RV dysfunction and failure in some diseases encountered at low-altitude. For example, acute RV dysfunction associated with HPV is common during exacerbation of respiratory diseases such as chronic obstructive pulmonary disease and diffuse fibrotic lung diseases (Weitzenblum, 1994; Judge et al., 2012; Swenson, 2013). Further, episodes of transient hypoxemia accompany sleep-disordered breathing. The most common type of sleep-disordered breathing is obstructive sleep apnea. HPV contributes to transient increases in PAP during sleep in obstructive sleep apnea patients (Kholdani et al., 2015). Taken together, acute hemodynamic load exerted on the RV due to HPV is common during exacerbation in diverse cardiopulmonary conditions and understanding of its pathophysiology may lead to the development of better management strategies.

Therapeutic approaches that could potentially improve RV performance upon acute high-altitude exposure should either reduce RV afterload or improve RV oxygenation. Decreased aerobic exercise capacity observed upon exposure to high-altitude is ascribed to both a decrease in arterial oxygen content and a limitation in maximal cardiac output (Fulco et al., 1998). Combined effects of several factors such as decreased blood volume, hypocapnia, increased blood viscosity, alterations of autonomic nervous system have been shown to limit maximal cardiac output (Wagner, 2000). A limitation in RV flow output due to HPV has also been shown to contribute to reduced maximal cardiac output and exercise capacity (Naeije et al., 2010). Indeed, several vasodilators such as sildenafil (Ghofrani et al., 2004; Richalet et al., 2005), bosentan (Faoro et al., 2009) and sitaxsentan (Naeije et al., 2010) have been shown to improve maximal exercise capacity along with decreasing PAP in humans upon acute high-altitude exposure. Sitaxsentan (Naeije et al., 2010), sildenafil (Reichenberger et al., 2007) and epoprostenol (Pavelescu and Naeije, 2012) also improved RV function in healthy volunteers upon acute high-altitude exposure or hypoxic breathing. Although, their direct impact on the RV cannot be excluded, these beneficial effects on the RV were probably due to lowering PAP and PVR. Taken together, vasodilators can improve RV function and exercise capacity at least in part due to reduction of RV afterload.

Another strategy to improve RV function is to improve RV oxygenation. Artificial oxygen carriers initially developed as “blood substitutes” are now considered also for other therapeutic concepts as “oxygen therapeutics” (Spahn, 2018). Recently, a beneficial effect of a novel cross-linked hemoglobin-based oxygen carrier was demonstrated in an experimental model of acute mountain sickness in rabbits and goats (Zhang et al., 2021). Application of a carbon monoxide-saturated hemoglobin-based oxygen carrier significantly improved cardiac performance in mice exposed to high-altitude (Wang et al., 2017). Furthermore, in lambs exposed to acute hypoxia, administration of a novel oxygen delivery biotherapeutic Omniox-cardiovascular was associated with preserved RV contractile function despite elevated PVR (Boehme et al., 2018). Thus, artificial oxygen carriers might be useful in improving RV function in conditions of acute hypoxia exposure. Similarly, agents improving cardiomyocyte tolerance to hypoxia may also help to maintain cardiac function and exercise capacity at high-altitude. For example, trimetazidine has been shown to prevent cardiorespiratory fitness impairment and improve left ventricular systolic function in healthy volunteers during acute high-altitude exposure (Yang et al., 2019). However, it remains unknown whether trimetazidine can improve RV function upon acute exposure to high-altitude.

## Limitations

Our systematic review has several limitations. As with any systematic review, publication bias and the possibility of missing potentially relevant articles cannot be excluded. However, we attempted to minimize such issues by using a robust search strategy including checking bibliography of original and review articles on the relevant topics.

In general, heterogeneity and small sample size limit the conclusions that can be drawn from the systematic analysis of the studies. Our systematic review identified considerable heterogeneity between study results. In the majority of the studies, RV function and morphology parameters were under-reported. Studies also differed significantly in terms of mode of altitude ascent. In studies with a staged acclimatization, participants spent nights at intermediate altitudes, while in other studies, subjects were brought to high-altitude by vehicles within a short time. In addition, results of acute altitude simulation studies cannot be fully extrapolated to the high-altitude field conditions. For example, reduction of total blood volume that occurs at high-altitude is not observed during acute simulated altitude (Stembridge et al., 2016). Moreover, the debate about the potential differences in modalities of hypoxia challenge remains unresolved (Savourey et al., 2003; Mounier and Brugniaux, 2012). Furthermore, echocardiography examinations were performed at different time-points of exposure to hypoxia. All these factors might have influenced the changes in the RV function parameters at a target altitude. Moreover, only very few studies considered other factors potentially influencing the RV response to acute hypoxia such as gender (Boos et al., 2016), age (Stewart et al., 2020) and ethnicity (Gaur et al., 2021).

With the advent of speckle-tracking echocardiography, detection of subtle cardiac abnormalities might have been improved. However, only in a few of the included studies such a sensitive technique was employed. In addition, in most of the studies, participants were middle-aged males; thus, our results probably are not applicable to other groups such as females and other age groups. Since we considered studies only on healthy subjects, our conclusions cannot be extended to individuals with underlying diseases. Conventional echocardiography and Doppler echocardiography are considered as less accurate non-invasive methods to assess pulmonary hemodynamics and cardiac function compared to invasive techniques (Fisher et al., 2009). Invasive methods, such as right heart catheterization, are less suitable due to ethical considerations in healthy individuals and challenging logistics in remote areas (Fagenholz et al., 2012; Feletti et al., 2018). Nevertheless, numerous studies have validated echocardiography by comparing it with invasive measurements of pulmonary hemodynamics at high-altitude (Allemann et al., 2000; Fagenholz et al., 2012).

Some studies included in the review reported similar number of subjects from the same authors, which might be the case that the same subject's data might have been used twice in two different publications. Although, we did not contact the authors regarding this issue, we have been very careful to screen the included studies if the authors have mentioned that the data presented in their studies had been used in previous publications.

## Conclusion

This systematic review demonstrated that the reports on the effects of acute exposure to hypoxia on the RV are controversial and inconclusive. This may be due to significantly different study designs and non-optimal reporting of multiple RV parameters as recommended by international guidelines. Moreover, the potential impact of other factors such as gender, age, mode of ascent and physical activity on RV responses to hypoxia largely remained neglected. Larger-scale studies and longer follow-up are needed to explore RV adaptation to hypoxia and associated increased afterload and its clinical relevance. Future research should study RV physiology by assessing multiple conventional echocardiography parameters along with the recently developed techniques, such as speck-tracking and 3D echocardiography. Thus, this comprehensive overview will promote reproducible research with improved study design and methods for the future large-scale prospective studies assessing RV function in accordance with the international guidelines, which eventually may provide important insights into the RV response to acute hypoxia exposure.

## Data Availability Statement

The original contributions presented in the study are included in the article/Supplementary Material, further inquiries can be directed to the corresponding author/s.

## Author Contributions

ArM, MS, NK, and AkpS conceived and designed the study, drafted the manuscript, and provided overall supervision. ArM, MS, NK, MD, AbM, and AkyS performed data acquisition, analysis, or interpretation. ArM, MS, NK, MD, AbM, AkyS, and AkpS critically revised important intellectual content in the manuscript and approved the final version of the manuscript. All authors contributed to the article and approved the submitted version.

## Funding

This work was supported by Ministry of education and science of the Kyrgyz Republic (No. 0005823).

## Conflict of Interest

The authors declare that the research was conducted in the absence of any commercial or financial relationships that could be construed as a potential conflict of interest.

## Publisher's Note

All claims expressed in this article are solely those of the authors and do not necessarily represent those of their affiliated organizations, or those of the publisher, the editors and the reviewers. Any product that may be evaluated in this article, or claim that may be made by its manufacturer, is not guaranteed or endorsed by the publisher.
