# Supplementary material for: Right Ventricular Response to Acute Hypoxia Exposure: A Systematic Review
Source: Front Physiol. 2022 Jan 12;12:786954. doi: 10.3389/fphys.2021.786954 (PMC8791628; doi:10.3389/fphys.2021.786954)
Supplement: Supplementary file 1 [file Table_1.DOCX]

**Literature search strategy**

***PubMed***

(01.01.2005-31.05.2021): N= 73

(("right ventric*"[All Fields] OR "RV function"[All Fields] OR "RV dysfunction"[All Fields] OR "cardiac function"[All Fields]) AND ("individual*"[All Fields] OR "subject*"[All Fields] OR "human*"[All Fields]) AND ("echocardiograph*"[All Fields] OR "ultrasound imaging"[All Fields]) AND (("hypoxi*"[All Fields] AND ("exposure"[All Fields] OR "exposure s"[All Fields] OR "exposured"[All Fields] OR "exposures"[All Fields] OR "exposuring"[All Fields])) OR "high altitude"[All Fields] OR ("hypoxi*"[All Fields] AND ("chamber"[All Fields] OR "chamber s"[All Fields] OR "chambered"[All Fields] OR "chambers"[All Fields])) OR ("hypoxi*"[All Fields] AND "room"[All Fields]))) AND ((humans[Filter]) AND (english[Filter]) AND (alladult[Filter]) AND (2004:2021[pdat]))

***Web of Science***

(01.01.2005-31.05.2021): N= 87

5

**((#3) AND #2) AND #1** and **Review Articles** (Exclude – Document Types) and **Associated Data** and **Physiology** or **Cardiac Cardiovascular Systems** or **Respiratory System** (Web of Science Categories) and **Book Chapters** (Exclude – Document Types) and **English** (Languages) and **Physiology** or **Cardiac Cardiovascular Systems** or **Respiratory System** (Web of Science Categories) and **2004** or **2003** or **2002** or **2001** or **2000** or **1999** or **1998** or **1997** or **1995** or **1994** or **1993** or **1992** or **1991** or **1990** (Exclude – Publication Years)

Edit

Add to Search

[87](https://www.webofscience.com/wos/woscc/summary/bb75dac7-662a-4324-9ae3-2b310d03afe8-00856426/relevance/1)

4

**((#3) AND #2) AND #1**

Edit

Add to Search

[278](https://www.webofscience.com/wos/woscc/summary/8141cbe4-8257-4f09-9d52-f1a68f3f16e1-00856100/relevance/1)

3

**ALL=("right ventric*" OR "RV function" OR "RV dysfunction" OR "cardiac function")**

Edit

Add to Search

[97,046](https://www.webofscience.com/wos/woscc/summary/b84c8896-4efa-4057-85d7-503ff734bc5f-008560ca/relevance/1)

2

**ALL=("hypoxi* exposure" OR "high altitude" OR "hypoxi* chamber" OR "hypoxi* room")**

Edit

Add to Search

[37,957](https://www.webofscience.com/wos/woscc/summary/5126d895-90dc-4fa5-a20d-0d0d6b7e69b0-00855dcc/relevance/1)

1

**ALL=(human* OR subject* OR individual*)**

Edit

Add to Search

[11,653,680](https://www.webofscience.com/wos/woscc/summary/e1170424-f144-472d-9b1d-d49e2080d29a-00855ba2/relevance/1)

**Query link**: https://www.webofscience.com/wos/woscc/summary/bb75dac7-662a-4324-9ae3-2b310d03afe8-00856426/relevance/1
